# Poster Session II - A197 TEMPORAL TRENDS IN ANTI-REFLUX MEDICATION USE IN VERY PRETERM INFANTS: A POPULATION-BASED COHORT STUDY

**DOI:** 10.1093/jcag/gwaf042.197

**Published:** 2026-02-13

**Authors:** S Tanner, H Stevens, L Morrison, M Higgins, S Ghotra

**Affiliations:** Dalhousie University Faculty of Medicine, Halifax, NS, Canada; Dalhousie University Faculty of Medicine, Halifax, NS, Canada; Dalhousie University Faculty of Medicine, Halifax, NS, Canada; Dalhousie University Faculty of Medicine, Halifax, NS, Canada; Dalhousie University Faculty of Medicine, Halifax, NS, Canada

## Abstract

**Background:**

Gastroesophageal reflux disease (GERD) affects up to 22% of infants born before 34 weeks’ gestation. Diagnosis is challenging and often relies on clinical judgment, as no standardized test can reliably rule out acid reflux. Management options include a trial of anti-reflux medications (ARMs) such as histamine-2 receptor antagonists (H2RAs) and proton pump inhibitors (PPIs). Recent guidelines discourage routine ARM use in pediatric population due to limited efficacy and potential health risks. Understanding prescribing patterns over time across both health care settings, in NICU and community, is key to reduce unnecessary ARM exposure in preterm infants.

**Aims:**

To describe temporal trends in ARM use during the first six months of life in very preterm infants.

**Methods:**

A retrospective population-based cohort study, using the provincial Perinatal Follow-Up Program database, was conducted. The study included all infants born at < 31 weeks gestational age between 2005 and 2023, excluding those with chromosomal or congenital anomalies, or neonatal deaths. Descriptive statistics and Chi-Square tests were used.

**Results:**

A total of 952 infants were enrolled. Of them, 445 (46.7%) infants were prescribed ARMs within the first six months of life (of these, 65.8% infants were prescribed in the NICU). Over the study period, ARM use decreased significantly but remained high at 35.1% in the last epoch. The prescription of H_2_ receptor antagonists, prokinetics, and combination therapies declined, whereas the use of proton-pump inhibitors rose over time (from 2.6% to 33.3%). The use of ARMs in the NICU decreased over time but remained common in community settings following NICU discharge.

**Conclusions:**

The continued frequent use of ARMs among very preterm infants remains concerning, given the contrast with recent clinical care guidelines. While the reduction in NICU-initiated ARM prescriptions indicates positive progress and guideline adherence, the ongoing high rates of post-discharge use suggest persistent gaps in continuity of care and knowledge translation. These findings highlight the need for targeted education for both NICU and community care providers to reduce unnecessary exposure and improve alignment with evidence-based practice.

A197 Table 1: Anti-reflux medication use in the first 6 months of life, over time, and by type. Infants grouped by birth year into 4 categories.

Funding Agencies: None

**Table:** 

Birth year	2005-2009 n = 269	2010-2014 n = 281	2015-2019 n = 237	2020-2023 n = 165	Total n = 952	p value
ARM use in first 6 months, n (%)	143 (53.2)	148 (52.7)	96 (40.5)	58 (35.1)	445 (46.7)	<0.001^*^
Exclusive PPI use, n (%)	7 (2.6)	23 (8.2)	63 (26.6)	55 (33.3)	148 (15.5)	<0.001^*^
Exclusive H2RA use, n (%)	90 (33.5)	87 (31.0)	23 (9.7)	3 (1.8)	203 (21.3)	<0.001^*^
Use of both PPI and H2RA, n (%)	46 (17.1)	38 (13.5)	10 (4.2)	0	94 (9.9)	<0.001^*^
Use of adjunct prokinetic+, n (%)	44 (16.4)	27 (9.6)	4 (1.7)	0	75 (7.9)	<0.001^*^
ARM started in NICU, n (%)	102 (37.9)	107 (38.1)	45 (19.0)	38 (23.0)	292 (30.7)	<0.01^*^
ARM started after discharge, n (%)	41 (15.2)	41 (14.6)	51 (21.5)	20 (12.1)	153 (16.1)	0.049^*^

+ Prokinetic medications were never used as a single agent.

* p-value < 0.05.

